# Prevalence and Clinical Correlates of Self-Harm Behaviors in Gilles de la Tourette Syndrome

**DOI:** 10.3389/fpsyt.2019.00638

**Published:** 2019-09-05

**Authors:** Natalia Szejko, Andrzej Jakubczyk, Piotr Janik

**Affiliations:** ^1^Department of Neurology, Medical University of Warsaw, Warsaw, Poland; ^2^Department of Bioethics, Medical University of Warsaw, Warsaw, Poland; ^3^Department of Psychiatry, Medical University of Warsaw, Warsaw, Poland

**Keywords:** Gilles de la Tourette syndrome, self-harm behavior, deliberate self-injury, unintentional self-injury, tics, compulsions

## Abstract

**Introduction:** Major symptoms of Gilles de la Tourette syndrome (GTS) are tics, but in 90% of cases, psychiatric comorbidities occur. Self-harm behaviors (SHBs) could result from deliberate action and unintentional injury from tics.

**Methods:** We examined 165 consecutive GTS patients aged 5 to 50 years (75.8% males). The median duration of GTS was 14 years (interquartile range, 9–22 years). The patients were evaluated for GTS and comorbid mental disorders according to the *Diagnostic and Statistical Manual of Mental Disorders, Fourth Edition, Text Revision*. Self-harm behavior was diagnosed during the interview. To determine a direct relationship between SHB and clinical variables, we conducted two analyses, at the time of evaluation and lifetime. We also compared the group of children and adults with SHB. We also tried to distinguish between deliberate (non–tic-related SHB) and accidental (tic-related SHB).

**Results:** Lifetime SHB was reported by 65 patients (39.4%), and in 55 of the cases, it was present at the time of evaluation. The age at the onset of SHB was reported in 55 of the cases (84.6%), and the median was 10 years (interquartile range, 7–13 years). In 30 of the patients (46.2%), SHB was evaluated as mild; in 26 (40%), as moderate; and in only 9 cases (13.9%), as severe. In the multivariable analysis for the predictor of lifetime SHB, attention-deficit/hyperactivity disorder (*p* = 0.016) and obsessive-compulsive disorder (OCD; *p* = 0.042) were determined as risk factors, while for current SHB, only tic severity (*p* < 0.0001) was statistically significant. When comparing predictors of SHB for children and adults, tic severity was determined as predictor for lifetime SHB in children (*p* < 0.0001), while the anxiety disorder was associated with lifetime SHB in adults (*p* = 0.05). Similarly, tic severity was a predictor of current SHB in the children group (*p* = 0.001), but this was not confirmed for adults. The group of patients with tic-related and non–tic-related SHB did not differ.

**Conclusions:** Self-harm behavior appears mostly in children and adolescents and rarely begins in adulthood. Self-harm behavior is associated mainly with tic severity, obsessive-compulsive disorder, and attention-deficit/hyperactivity disorder. Clinical correlates of SHB are age related and differ at different points of life. Tic severity is the main factor associated with SHB in children. In the adult group, anxiety disorder and other psychiatric comorbidities may play the most important role.

## Introduction

Gilles de la Tourette syndrome (GTS) is a neurodevelopmental disorder affecting children and adults. To diagnose GTS, numerous motor tics as well as at least one vocal tic should be present during a minimum period of 1 year. The onset must be in the childhood. The major symptom is tics, defined as sudden, rapid, recurrent, nonrhythmic motor movements or vocalizations. In 90% of cases of GTS, psychiatric comorbidities occur, most commonly attention-deficit/hyperactivity disorder (ADHD) and obsessive-compulsive disorder (OCD) (1).

Deliberate, purposeful, nonaccidental, and repetitive infliction of self-harm is defined differently in the literature, as self-injury, self-inflicted violence, nonsuicidal self-injury (NSSI) (2), or self-injurious behavior (SIB). These terms are used synonymously, and all of them refer to an adverse infliction of injury. For the purpose of our study, we have opted to use the term self-harm behavior (SHB), which includes both deliberate and inadvertent self-injury. The lifetime prevalence of SHB in the general population varies from 5.9% to even 49% (3, 4). The incidence of SHB in GTS is estimated to vary between 4% and 53% according to different studies, which could depend on the definition of self-injury (1, 2). Higher rates of SHB are probably also related to the inclusion of subjects with inadvertent self-directed behavior caused by the tics. Because of noncompliance with the definitions, the particular movement may be misinterpreted. For example, head-banging that leads to self-injury could be classified either as deliberate behavior or as an involuntary action (tic) if the intentionality of the movement had not been assessed. Differentiation between intentional and nonintentional SIB is often difficult, and in some patients, it is not easy or even impossible to classify a particular behavior as a tic, compulsion, or SHB. Therefore, we decided to combine intentional and unintentional self-injury resulting from tics or compulsions in SHB, without categorising the intentionality and phenomenology of these behaviors.

Previous studies about self-injury in GTS refer to various aspects of this phenomenon. Mathews et al. (2) focused on the lifetime prevalence of SHB. The authors analyzed clinical data of nearly 300 patients with GTS and divided those who suffered from self-injury into mild, moderate, and severe cases. As a result, mild/moderate self-injury correlated with the presence of obsessive and compulsive symptoms such as aggressive obsessions or violent or aggressive compulsions and with the presence of OCD and overall number of obsessions. Severe self-injury in GTS was associated with episodic rages and risk-taking behaviors. Both mild/moderate and severe self-injuries were also correlated with tic severity. Another study, which mentioned self-injury only briefly, along with a number of other clinical variables, was by Sambrani et al. (1). The incidence of self-injury was 39.4%, and it was highly associated with complex motor tics and coprophenomena. Just recently, Chen et al. (5) published article about increased risk of traumatic brain injury in GTS. The authors used the National Health Insurance Research Database of Taiwan and compared 2261 GTS patients and 20,349 non-GTS controls matched by gender and age between 2000 and 2012 and followed until the end of 2013. The results showed that violent motor tics or severe SHBs were the most important predictors of brain injury in GTS. The authors concluded that GTS patients with ADHD, OCD, or depressive disorder had a higher traumatic brain injury incidence rate, while the use of antipsychotics was a protective factor. Kano et al. (6) have evaluated comorbidities in 88 Japanese GTS patients and found significantly higher rates of SHB in GTS patients with psychiatric comorbidities (OCD and ADHD). Similar study was conducted by Eapen et al. (7), who included 91 consecutive adult GTS subjects. In this study, SHB occurred in 43.9% of cases, and obsessive-compulsive symptoms (OCS) correlated positively with ADHD and SHB.

The term “malignant GTS” was introduced by Cheung et al. (8). Malignant GTS was defined as two or more emergency room visits or one or more hospitalizations for GTS symptoms or its associated behavioral comorbidities. Of 333 GTS patients evaluated in this study, during the 3-year period, 17 (5.1%) met the criteria for malignant GTS. Hospital admission or emergency room visits were for tic-related injuries, SIB, uncontrollable violence and temper, and suicidal ideation/attempts. Compared with patients with nonmalignant GTS, those with malignant GTS were significantly more likely to have a personal history of OCS/OCD, complex phonic tics, coprolalia, copropraxia, SHB, mood disorder, suicidal ideation, and poor response to medications. Malignant GTS was associated with greater severity of motor symptoms and the presence of two or more behavioral comorbidities. Cheung et al. (8) showed that OCD/OCS in particular may play a central role in malignant GTS and were associated with life-threatening tics, SHB, and suicidal ideation.

There are also a number of case reports describing malignant GTS. Fasano and Galluccio (9) published recently the article dedicated to brain injury secondary to severe tics. The authors reported about a 17-year-old male patient with malignant GTS who developed severe head tics that led to head trauma and subdural and subarachnoid haemorrhage followed by massive edema managed with decompressive craniotomy. Krüger and Müller-Vahl (10) published a case report of malignant GTS with self-extraction of teeth. Hood et al. (11) reported about severe self-inflicted oral lacerations in GTS. However, such extreme cases are rare and (as mentioned above) were mainly reported in case reports. In general, extremely severe SHB is seldom and was reported to occur in only 4% to 5% of GTS cases (2, 8).

As all previous publications have only taken into account deliberate and lifetime self-injury, we wanted to extend current knowledge and investigate both the lifetime and additionally current SHB, as only associations with current clinical characteristics reflect the actual relationship between the two variables. As mentioned earlier, we aimed at assessing the prevalence and clinical correlates of all SHBs (deliberate and inadvertent). We hypothesized that SHBs would be associated with psychiatric comorbidities (ADHD, OCD, anxiety, and depression) and complex motor tics, coprophenomena, and tic severity.

In addition, we wanted to investigate and compare predictors of lifetime and current SHB in children and adults with GTS. All previous studies reported only about lifetime SHB as well as never distinguished separately the results for children and adults. Moreover, we attempted to distinguish between tic-related and non–tic-related SHB as we predicted that it could be correlated with different clinical factors. As those phenomena have never been studied from that point of view, the study had an exploratory character.

## Materials and Methods

### Study Participants and Procedures

The cohort of GTS cases consisted of 165 consecutive ambulatory patients aged 5 to 50 years [median, 14 years; interquartile range (IQR), 9–22 years; 125 males (75.8%)]. The subjects were evaluated from 2013 to 2018. In total, 100 children [60.6%; median, 10 (IQR, 8–12.5) years] and 65 adults [39.4%; median, 25 (IQR, 21–32) years] were enrolled. The median age at the onset of tics was 6 years (IQR, 5–7 years). The median of disease duration was 7 years (IQR, 4–13 years), 4 years (IQR, 2–7 years) in children and 18.5 years (IQR, 12–23 years) in adults; 136 (82.4%) of the patients had at least one psychiatric comorbidity. General characteristics of GTS patients are presented in **Table 1**. The patients were evaluated for the clinical diagnosis of GTS and comorbid mental disorders according to the *Diagnostic and Statistical Manual of Mental Disorders, Fourth Edition, Text Revision*. Obsessive-compulsive symptoms were diagnosed if obsessions and compulsions were egosyntonic, in contrast to egodystonic symptoms that characterize OCD (12). Obsessive-compulsive symptom is never impairing and never requires treatment, while OCD requires intervention. We used significant social skill problems as the principal feature of autistic traits because only a few patients had been diagnosed with autism spectrum disorder in our cohort. To assess the current tic severity, we used the Yale Global Tic Severity Scale (YGTSS) (13, 14). All the patients were examined and evaluated by the same clinician experienced in movement disorders (PJ). The study was designed as a one-time registration study, and no new clinical data obtained in follow-up visits were included in the analysis. We used diﬀerent methods of data collection for children and adults. Most of the clinical information regarding children was provided by their parents, whereas adults reported it themselves. All questions asked during the interview were part of a routine practice, and therefore we do not report refusal rate in this study.

**Table 1:** Psychiatric comorbidities in children, adolescents, and adults with GTS.

Comorbid mental disorder	Children (n = 75)	Adolescents (n = 25)	Adults (n = 65)
ADHD	28%, n = 21	20%, n = 5	23.1%, n = 15
OCD	6.7%, n = 5	8%, n = 2	33.8%, n = 22
OCS	42.7%, n = 32	52%, n = 13	47.7%, n = 31
Depression	0%, n = 0	12%, n = 3	33.8%, n = 22
Anxiety disorder	42.7%, n = 32	56%, n = 14	50.8%, n = 33
Aggression	37.3%, n = 28	28%, n = 7	36.9%, n = 24
Significant social skill problems	14.7%, n = 11	20%, n = 5	16.9%, n = 11

GTS, Gilles de la Tourette syndrome; ADHD, attention-deficit/hyperactivity disorder; OCD, obsessive-compulsive disorders; OCS, obsessive-compulsive symptoms.

Lifetime prevalence is shown. Groups are defined as children (aged 5–12 years), adolescents (aged 13–18 years), and adults (aged >18 years).

### Definition of SHB

During the interview, the patients were asked if they had experienced self-harm and whether they could define its intentionality. Particularly, we asked whether they had this symptom in the past as a consequence of abnormal movements or deliberate action, whether these behaviors were considered entirely involuntary or if they had control over them, and at what age the SHB started. However, many patients could not clearly define the intentionality of the SHB, and often it was difficult to classify these complex behaviors as tics, compulsions, or deliberate action due to symptom overlapping. Nevertheless, we decided to divide SHB in tic-related and non–tic-related according to criteria elucidated in the section *Types of SHB* of the manuscript.

The patients were also questioned about the type of injury that had followed the movement, whether or not the SHB left a visible mark on the skin and if it required any medical attention. The SHB in our study manifested as skin picking, self-pinching, wound scratching, head banging, self-hitting, self-biting (nail, hand, or cheek), injury to the teeth because of grinding, accidental self-mutilation, or burning due to touching hot objects.

With regard to intensity, we divided SHB into mild, moderate, and severe. Mild SHB consists of temporary self-harm that does not interfere with everyday functioning, is not reported spontaneously either by the patient or by the family, and does not leave any visual, objectively perceptible signs. Moderate SHB, on the other hand, causes tissue damage, but it is superficial and does not require any additional medical help. Moderate SHB can therefore lead to bruises or wounds, it causes physical pain and potentially may lead to more severe injuries (e.g. hitting oneself with a glass bottle or pushing eyeballs with fingers). Finally, severe SHB leads to significant psychological and physical impairment and normally requires additional medical intervention (e.g. broken bones, wounds that need suturing, lesions that cause severe disability or permanent body deformation).

### Types of SHB

Despite the difficulties mentioned earlier, we attempted to divide SHB into tic-related (accidental) and non–tic-related (deliberate) group. We defined tic-related as self-hitting, while other types of SHB, such as biting, wound scratching, self-injury that leads to wounds, or skin picking or pinching, were considered as non–tic related. Duration of SHB was an important criterion to make this distinction. Tic-related self-hitting was always brief and sudden in contrast to deliberate SHB that was longer and less violent. Sometimes, self-hitting occurred in series, but each movement was very brief, similar to tics. What is more, normally patients described self-hitting as the consequence of tics and therefore accidental movement. Some SHBs that resembled compulsions, as they were more complex, purposeful, prolonged, and directed to reduce inner tension, were included into non–tic-related SHB.

Here we summarize all types of SHB that occurred in our group:

1. Tic related
  self-hitting
2. Non–tic related
  - skin picking
  - self-pinching
  - wound scratching
  - head banging
  - self-biting (nails, hand, or cheek)
  - teeth injury because of grinding
  - deliberate self-mutilation
  - burning due to touching of hot objects

### Lifetime SHB Versus Current SHB

We conducted two comparative analyses. The first included the group of patients without any history of SHB (SHB−) and the group of patients with a history of SHB in the past or at the time of evaluation (SHB+). When categorizing patients as having SHB at the time of evaluation, we took into account last 1 week. In the second comparative analysis, we divided all the patients into two groups: SHB current+ (GTS patients with SHB at the time of evaluation) and SHB current– (GTS patients without SHB within last week). As a result, SHB current− included both patients without any history of SHB and those with SHB in the past but not at the time of evaluation. Moreover, in this second analysis, we took into account only SHB and other variables present at the time of the examination, as we wanted to determine whether there was a direct relationship between the demographic and clinical parameters and the SHB. Finally, we wanted to compare the two analyses and determine whether the factors that influence the lifetime prevalence of SHB in GTS differ from those that are related to the SHB at the time of examination. For example, a lifetime diagnosis of depression does not necessarily contribute to the occurrence of SHB as both symptoms could have occurred at different moments in the lifetime. The exact relationship could be determined only when both symptoms are present at the same time. As part of lifetime and current comparison, we also have done separate analysis for children and adults as we suspected that those results could differ.

### Statistical Analysis

The statistical analyses were performed using STATISTICA version 13.1 (Statsoft Inc. Palo Alto, CA, USA) and SPSS version 25 (IBM Corp, Armonk, NY) software. The normality of distribution was assessed using the Shapiro-Wilk test. For the parametric variables, data are presented as arithmetic means and SDs (mean ± SD). For the nonparametric variables, data are presented as median and quartiles (25th, 75th). The categorical variables are presented as frequencies (percentages). Parametric data were compared using an independent *t* test and the nonparametric data using the Mann-Whitney *U* test, as appropriate; the categorical data were compared using Fisher exact test (two-sided).

In both the analyses, the comparisons between the groups were considered significantly different when the two-tailed test was *p* < 0.05. All the variables that were significant in the primary analyses were entered into a logistic regression analysis in order to determine the risk factors for the lifetime or current SHB in GTS patients. In addition, gender and age were entered into the multivariate model as control variables.

When analyzing predictors for lifetime and current SHB in children and adults, we used the same statistical methods as mentioned above. Therefore, all variables that were found significant in univariate analysis were included in the multivariate analysis.

## Results

### General Characteristics

Lifetime SHB was reported by 65 patients (39.4%), whereas in 55 of the cases, it was present currently. The prevalence of SHB did not differ between adults and children: 41.5% (27/65) and 38% (38/100), respectively (*p* = 0.65).

Age at the onset of SHB was known in 55 of the cases, and the median value was 10 years (IQR, 7–13 years). In 28 of the cases, SHB had started during childhood (before 11 years of age); in 22 of the cases, during adolescence (12–18 years); and in five of the cases, in adulthood. In 30 of the patients (46.2%), SHB was evaluated as mild; in 26 (40.0%), as moderate; and in 9 cases (13.9%), as severe. The most severe cases included teeth grinding (bruxism), leading to damage to the enamel, and hitting the head that caused damage to the teeth. Another patient was operated on several times due to elbow hitting, whereas another case of self-hitting led to the patient breaking his/her nose. One female patient ended up blind in one eye and with a severe impairment in the other eye as a result of punching herself in the eyes.

The most frequent types of SHB were as follows: self-hitting (n = 44), self-biting (n = 29), and scratching, especially of wounds (n = 20); 26 patients had only one SHB, 22 patients suffered from two types of SHB, and 17 had three or more.

### Lifetime Versus Current SHB

In the comparative analysis of lifetime SHB+ and SHB− groups, there were no differences in age and gender distribution. Nevertheless, patients with SHB had more severe tics and suffered more frequently from psychiatric comorbidities (**Table 2**). The variables that were significantly associated with lifetime SHB in the univariate analyses (and therefore were included in the multivariate analysis) were as follows: YGTSS, depression, ADHD, OCD, anxiety disorder, aggression, and significant social skill problems. In the multivariate logistic regression, only ADHD and OCD remained significant (**Table 3**). The model was significant (*p* = 0.001, χ^2^ = 30.436, and *R* ^2^ Nagelkerke = 0.602).

**Table 2:** Comparison of lifetime SHB− and SHB+ groups.

	All GTS patients			Children with GTS			Adults with GTS		
	SHB+ (n = 65)	SHB− (n = 100)	*p*	SHB+ (n = 38)	SHB− (n = 62)	*p*	SHB+ (n = 27)	SHB− (n = 38)	*p*
Age at evaluation [years] [median] (IQR)	14 (11-22)	11 (9-23)	0.28	11.5 (9-14)	9.5 (8-11)	**0.009**	27 (20-33)	25 (22-32)	0.904
Gender (male/female)	50/15	75/25	0.778	30/8	47/15	0.717	20/7	28/10	0.972
YGTSS [median] (IQR)	61 (46-72)	35 (24-51)	**0.01**	60.5 (46–40)	29.5 (19–41)	**0.000**	71 (46–79)	44.5 (33–60)	**0.0017**
Depression	n = 17 (26.2%)	n = 8 (8.0%)	**0.002**	2 (5.3%)	1 (1.6%)	0.299	n = 15 (55.6%)	n = 7 (18.4%)	**0.002**
ADHD	n = 25 (38.5%)	n = 16 (16,0%)	**0.001**	n = 14 (36.8%)	n = 12 (19.4%)	0.053	n = 11 (40.7%)	n = 4 (10.2%)	**0.004**
OCD	n = 18 (27.7%)	n = 11 (11.0%)	**0.006**	n = 6 (15.8%)	n = 1 (1.6%)	**0.007**	n = 12 (44.4%)	n = 10 (26.3%)	0.128
OCS	n = 34 (52.3%)	n = 42 (42.0%)	0.194	n = 21 (55.3%)	n = 24 (38.7%)	0.106	n = 13 (48.1%)	18 (47.4%)	0.951
Anxiety disorder	n = 41 (63.1%)	n = 38 (38.0%)	**0.002**	n = 20 (52.6%)	n = 26 (41.9%)	0.298	n = 21 (77.8%)	n = 12 (31.6%)	**0.0002**
Aggression	n = 34 (52.3%)	n = 25 (25.0%)	**0.0003**	n = 19 (50%)	n = 16 (25.8%)	**0.014**	n = 15 (55.6%)	n = 9 (23.7%)	**0.009**
Significant social skill problems	n = 17 (26.2%)	n = 10 (10.0%)	**0.006**	n = 12 (31.6%)	n = 4 (6.5%)	**0.0009**	n = 5 (18.5%)	n = 6 (15.8%)	0.772

SHB, self-harm behavior; GTS, Gilles de la Tourette syndrome; YGTSS, Yale Global Tic Severity Scale; ADHD, attention-deficit/hyperactivity disorder; OCD, obsessive-compulsive disorder; OCS, obsessive-compulsive symptoms; IQR, interquartile range.

Lifetime prevalence of psychiatric symptoms and disorders is shown. For nonparametric variables, data are presented as median and interquartile range. Categorical variables are presented as frequencies (percentages).

The bolded texts refers to the p value lower than 0.05.

**Table 3:** Logistic regression analysis for the predictors of lifetime SHB in GTS.

	All GTS patients		Children with GTS		Adults with GTS	
	OR (95% CI)	*p*	OR (95% CI)	*p*	OR (95% CI)	***p***
Age	1.070 (0.929-1.232)	0.349	1.143 (0.959-1.363)	0.136	1.012 (0.926-1.106)	0.794
Gender	1.028 (0.093-11.319)	0.982	2.066 (0.558-7.643)	0.277	0.902 (0.202-4.025)	0.892
YGTSS	1.061 (0.994-1.132)	0.074	1.062 (1.030-1.095)	**0.000**	1.092(0.985-1.054)	0.268
ADHD	26.369 (1.819-381.991)	**0.016**	—	—	3.759 (0.774-18.254)	0.101
OCD	39.330 (1.133-1365.095)	**0.042**	2.350 (0.200-27.624)	0.497	—	—
Anxiety disorder	6.893 (0.651-72.958)	0.109	—	—	4.016 (1.001-16.106)	**0.050**
Depression	10.514 (0.693-159.522)	0.090	—	—	2.555(0.606-10.755)	0.201
Agression	2.139 (0.284-1.20)	0.460	1.283 (0.390-4.219)	0.681	2.163 (0.577-8.105)	0.252
Significant social skill problems	0.207 (0.018-2.448)	0.212	2.182 (0.454-10.478)	0.330	—	—

SHB, self-harm behaviour; GTS, Gilles de la Tourette syndrome; YGTSS, Yale Global Tic Severity Scale; ADHD, attention-deficit/hyperactivity disorder; OCD, obsessive-compulsive disorder; OR, odds ratio; CI, confidence interval.

Lifetime prevalence of psychiatric symptoms and disorders is shown.

In the comparative analysis of the SHB current+ and SHB current− groups, there were also no differences in age and gender distribution. The vast majority of the factors that were significant for lifetime SHB in GTS were also detected for the SHB current+ group; those patients also had higher results on the YGTSS and were more commonly diagnosed with ADHD, OCD, and anxiety disorder and more frequently demonstrated aggressive behavior, as well as social skill problems, than the SHB current− group. Finally, the following variables were significantly associated with current SHB in univariate analyses and were included in logistic regression model: YGTSS, OCD, ADHD, anxiety disorder, depression, aggression, and significant social skill problems. The characteristics of current SHB are shown in **Table 3**. In the multivariate logistic regression for the SHB current+ group, only the YGTSS remained significant (**Table 5**). The model was significant (*p* = 0.000, χ^2^ = 58.282, and *R* ^2^ Nagelkerke = 0.413). We included only OCD in this analysis as OCD + OCS is also determined by the presence of OCD.

### Children Versus Adults With SHB

General characteristics of both adults and children are presented in **Table 1**. In children, the following variables were significantly associated with lifetime SHB in univariate analyses and were later included in logistic regression model: age, YGTSS, aggression, OCD, and significant social skill problems. In adults, univariate analyses showed significant associations between lifetime SHB and the following variables (which were later put in multivariate analysis): YGTSS, depression, ADHD, anxiety disorder, and aggression.

In multivariate analyses, tic severity was determined as predictor for lifetime SHB in children (*p* < 0.0001), while the anxiety disorder was associated with lifetime SHB in adults (*p* = 0.05).

In children, the following variables were significantly associated with current SHB in univariate analyses and were later included in logistic regression model: age, YGTSS, ADHD, OCD, OCS, anxiety disorder, aggression, and significant social skill problems. In adults, univariate analyses showed significant associations between current SHB and the following variables (which were later put in multivariate analysis): age, YGTSS, OCD, aggression, and social skills problems.

In multivariate analysis for predictors of current SHB, tic severity was a predictor of current SHB in the children group (*p* = 0.001); however, none of the variables reached statistical significance in logistic regression for predictors of current SHB in adults. For details, see **Tables 2**, **3**, **4**, and **5**.

**Table 4:** Comparison of SHB+ and SHB− current groups.

	All GTS patients			Children with GTS			Adults with GTS		
	SHB current+ (n = 55)	SHB current− (n = 110)	*p*	SHB current+ (n = 34)	SHB current− (n = 66)	*p*	SHB current+ (n = 21)	SHB current− (n = 44)	*p*
Age at evaluation [years] [median] (IQR)	14 (11-20)	12 (9-23)	0.21	11.5 (9–11)	10 (8–11)	**0.000**	22 (20–33)	25.5 (22-32)	**0.000**
Gender (male/female)	42/13	83/27	0.898	28/6	49/17	0.361	14/7	34/10	0.363
YGTSS [median] (IQR)	64 (51-76)	35 (23-53)	**0.000**	60.5 (50–70)	30.5(19-43)	**0.000**	71 (58–79)	44.5 (33–60)	**0.000**
Depression	n = 4 (7.3%)	n = 4 (3.6%)	0.305	n = 2 (5.9%)	n = 0 (0%)	0.047	n = 2 (9.5%)	n = 4 (9.1%)	0.955
ADHD	n = 18 (32.7%)	n = 17 (15.5%)	**0.011**	n = 13 (38.2%)	n = 12 (18.2%)	**0.028**	n = 5 (23.8%)	n = 5 (11.4%)	0.193
OCD	n = 13(23.6%)	n = 7 (6.4%)	**0.00135**	n = 5 (14.7%)	n = 1(1.5%)	**0.009**	n = 8 (38.1%)	n = 6 (13.6%)	**0.025**
OCS	n = 25(45.5%)	n = 43 (39.1%)	0.434	n = 19 (55.9%)	n = 23 (34.8%)	**0.044**	n = 6 (28.6%)	n = 20 (45.5%)	0.194
Anxiety disorder	n = 32 (58.2%)	n = 38 (34.6%)	**0.004**	n = 21 (61.8%)	n = 25 (37.9%)	**0.023**	n = 11 (52.4%)	n = 13 (29.5%)	0.074
Aggression	n = 26 (47.3%)	n = 29 (26.4%)	**0.00005**	n = 16 (47.1%)	n = 12 (18.2%)	**0.002**	n = 10 (47.6%)	n = 7 (15.9%)	**0.007**
Significant social skill problems	n = 16 (29.1%)	n = 5 (4.6%)	**0.00001**	n = 11 (32.4%)	n = 3 (4.5%)	**0.0002**	n = 5 (23.8%)	n = 2 (4.5%)	**0.019**

SHB, self-harm behavior; GTS, Gilles de la Tourette syndrome; YGTSS, Yale Global Tic Severity Scale; ADHD, attention-deficit/hyperactivity disorder; OCD, obsessive-compulsive disorder; OCS, obsessive-compulsive symptoms; IQR, interquartile range.

Current prevalence of psychiatric symptoms and disorders is shown.

The bolded texts refers to the p value lower than 0.05.

**Table 5:** Logistic regression analysis for the predictors of current SHB in GTS.

	All GTS patients		Children with GTS		Adults with GTS	
	OR (95% CI)	*p*	OR (95% CI)	*p*	OR (95% CI)	*p*
Age	0.980 (0.932-1.030)	0.417	1.131 (0.935-1.370)	0.205	0.969 (0.888-1.057)	0.476
Gender	1.218 (0.458-3.242)	0.693	0.985 (0.247-3.931)	0.983	1.657 (0.366-7.499)	0.512
YGTSS	1.055 (1.029-1.082)	**<0.0001**	1.052 (1.020-1.085)	**0.001**	—	—
ADHD	0.991 (0.349-2.816)	0.986	1.473 (0.385-5.638)	0.572	—	—
OCD	1.260 (0.346-4.580)	0.726	2.868 (0.169-48.734)	0.466	1.690 (0.353-8.091)	0.511
Anxiety disorder	1.741 (0.741-4.093)	0.204	1.229 (0.390-3.876)	0.725	—	—
Aggression	1.270 (0.474-3.460)	0.634	1.210 (0.296-4.951)	0.791	1.980 (0.461-8.502)	0.358
Significant social skill problems	3.393 (0.910-12.646)	0.069	2.629 (0.424-16.303)	0.299	2.515 (0.290-21.828)	0.403

SHB, self-harm behaviours; GTS, Gilles de la Tourette syndrome; YGTSS, Yale Global Tic Severity Scale; ADHD, attention-deficit/hyperactivity disorder; OCD, obsessive-compulsive disorder; OR, odds ratio; CI, confidence interval.

The bolded texts refers to the p value lower than 0.05.

### Tic-Related Versus Non–Tic-Related SHB

Forty-two patients (64.5%) had tic-related SHB, whereas 48 (73.8%) had non–tic-related SHB; in 25 patients (38.5%), both types of SHB occurred. We present only lifetime results, as we do not distinguish between current and lifetime results in this case. When comparing the patients with tic-related and non–tic-related SHB, no differences were found (**Table 6**).

**Table 6:** Comparison of tic-related and non–tic-related SHB.

	All patients with SHB		*p*
	Tic related (n = 17)	Non–tic related (n = 23)	
Age at evaluation [years] [median] (IQR)	13.5 (8-22)	15 (12-22)	0.408
Gender (male/female)	15/2	15/8	0.251
Children/adults	5/12	13/10	0.662
YGTSS [median] (IQR)	33 (0-73)	46 (15-71)	0.999
Depression	n = 3 (17.6%)	n = 6 (26.1%)	0.819
ADHD	n = 9 (52.9%)	n = 6 (26.1%)	0.222
OCD	n = 4 (23.5%)	n = 5 (21.7%)	0.991
OCS	n = 12 (70.6%)	n = 10 (43.5%)	0.234
Anxiety Disorder	n = 10 (58.8%)	n = 16 (69.6%)	0.780
Aggression	n = 8 (47.1%)	n = 11 (47.8%)	0.999
Significant social skill problems	n = 4 (23.5%)	n = 6 (26.1%)	0.983

SHB, self-harm behavior; GTS, Gilles de la Tourette syndrome; YGTSS, Yale Global Tic Severity Scale; ADHD, attention-deficit/hyperactivity disorder; OCD, obsessive-compulsive disorder; OCS, obsessive-compulsive symptoms; IQR, interquartile range. Lifetime prevalence of psychiatric symptoms and disorders is shown. For nonparametric variables, data are presented as median and interquartile range. Categorical variables are presented as frequencies (percentages). Patients with both types of SHB were excluded from the analysis.

## Discussion

Our study further confirms that SHB is an important phenomenon in GTS. To the best of our knowledge, all previous reports have been based on information about the lifetime symptom prevalence. They also did not include the separate analysis of children and adult group or intend to distinguish between deliberate and accidental SHB. For this reason, it was not possible to assess whether deliberate, non–tic-related SHB or inadvertent, tic-related SHB were directly related to the presence of OCD, ADHD, or tics. We investigated both the lifetime and the current prevalence of SHB. Therefore, we believe that our findings better reflect an actual relationship between SHB and other clinical factors. To give an example, when only lifetime SHB is considered in an adolescent, one incident of SHB at the age of 8 years could be, by mistake, interpreted as the result of OCD that started at the age of 10 years.

The lifetime prevalence of SHB in our group was 39.4%, which approximates to the values reported by other researchers for this symptom in GTS. Sambrani et al. (1) actually showed the same prevalence of 39.4% for lifetime SHB in their large study sample including 1032 GTS patients. Similarly, high prevalence of SHB in GTS of 43.9% was determined by Eapen et al. (7). Meanwhile, extremely severe SHB named as malignant GTS is seldom and was reported to occur in only 4% to 5% of GTS cases (2, 8). This may suggest that previous studies dedicated to self-injury in GTS also included patients with different behaviors related to either tics or comorbidities. None of the authors of the studies intended to distinguish between tic-related and non–tic-related SHB and included both deliberate as well as involuntary behaviors in their study. We have intended to do so, but failed to demonstrate any differences between those two groups. This could mean that there is an overlap between tic-related and non–tic-related SHB, and it is almost impossible to distinguish between them even for the patients themselves. Small groups of patients with particular psychiatric comorbidity may underline this finding as well. We found ADHD and OCD to be mostly associated with SHB in the whole group of GTS patients, but if we look at **Table 6**, we will find that ADHD and OCD groups consisted of less than 10 patients. We can only speculate that inclusion of larger groups could lead to different results, more similar to those obtained for all GTS patients. Finally, we probably did not establish clear and reliable criteria that could enable us to differentiate between tic-related and non–tic-related SHB as more than one-third of our patients reported both types of SHB. On the other hand, we cannot exclude that different types of SHB may have different etiology in the same patient.

The age at onset in our group for the majority of cases was in childhood and adolescence and only in a few cases in adulthood. Moreover, recall bias regarding the occurrence of SHB in childhood should be taken into account. For this reason, we can speculate that onset of SHB in adulthood can be even less often. This finding about SHB onset is consistent with reports about SHB in other diseases and in a healthy population (15–17). Interestingly, the average age at onset was 11 years, and this coincides with the worst tic severity period, which usually happens between 10 and 12 years of age (18). From the positive correlation of SHB with YGTSS score, we can speculate that severe tics may be associated with SHB risk or SHB could substantially contribute to tic severity. Thus, it is possible that SHB may add significantly to the impairment caused by tics.

Taking into account the severity of the SHB, in comparison to the study by Mathews et al. (2), our group had more patients with severe SHB. This could be due to referral bias as all of our patients were evaluated by the movement disorders specialist. That is why we assume they could have suffered from more severe tics. The patients with more severe mental diseases were likely to seek psychiatric advice. Interestingly, similarly to the study by Mathews et al., our sample showed that the most frequent types of self-harm could resemble complex tics or compulsions: self-biting, self-hitting, and scratching. This confirms the finding that OCD could be a risk factor for SHB and that auto lesions could, in fact, be the manifestation either of tics or of compulsions that could be present in GTS. In our analysis, the most important risk factor for SHB at the time of evaluation was tic severity, whereas lifetime SHB was first associated with comorbid psychiatric disorders, mainly ADHD and OCD. At this point, we have to remember that YGTSS provides information about tic severity during the last week and that tic intensity is rated automatically as 5 when self-harm is present: this could be one possible explanation for the current SHB association with tic severity. There is no objective method to measure the intensity of tics in the past; thus, for lifetime SHB, no measure for this factor could be used. One of the possible reasons that explain the differences between current and lifetime SHB is that some of the diagnoses (ADHD, OCD) and symptoms could be only temporarily present. For example, up to 85% of ADHD patients report symptom remission in adulthood (19). On the other hand, the onset of other disorders, for example, the onset of OCD, is normally at the age of 10 years or even later, so some patients may not have developed their symptoms yet. Our results also suggest that patients with SHB are more likely to suffer from anxiety and depressive symptoms. Moreover, anxiety disorder was determined as the risk factor for lifetime SHB in the group of adults with GTS. This findings stays in line with previous studies where the presence of depression or anxiety was an independent risk factor for different types of SHB (20–22). Depression was also a risk factor for SHB in patients with OCD, which coexists frequently in GTS (23). Also, Chen et al. (5) reported that depression was one of the risk factors for SHB. Meanwhile, Kano et al. (6) identified OCD and ADHD as predictors for SHB, and Eapen et al. (7) found only the relationship with OCS.

When comparing the results for children and adults, it seems that children could have more tic-related SHB as the risk factor for both lifetime and current SHB was tic severity. On the other hand, the only risk factor for adults was anxiety disorder, indicating that in older patients psychiatric comorbidities could play a more important role compared to tic severity in the development of SHB. Surprisingly, neither OCD nor ADHD that correlated with lifetime SHB in the whole group of GTS patients was found to be related to SHB when taken into account children and adults as separate groups in multivariate analyses (**Tables 3** and **5**). A small group of patients with particular psychiatric disorder may underlie this apparent discrepancy. Attention-deficit/hyperactivity disorder and OCD in SHB+ patients were more frequent compared to the SHB− group (**Table 2**). It is plausible that the results were biased because the groups were too small, and that is why the results did not reach statistical significance. Nevertheless, further studies are needed to investigate risk factors of SHB in separate groups of children and adults.

Although our study does not answer the question why patients with GTS engage in SIBs, it provides some clues to understand it. Based on the current literature, NSSI most commonly temporarily alleviates the overwhelming negative emotions (24). The same is true with regard to tics that sometimes “are performed” by the patients in response to unpleasant, preceding premonitory urges to neutralize them and reach relief. In this context, tics might be considered as not entirely involuntary movements, and both tics and NSSI are used to neutralize negative emotions. Regarding OCD, compulsions are defined as urge-driven complex behaviors aimed to reduce anxiety or distress. Similarly to NSSI and tics, they serve to reduce negative and unpleasant emotions. Strong association between SHB, tic severity, and OCD observed in our study provides an evidence that SHB, tics, and compulsions may be a part of the same complex behavioral spectrum. Attention-deficit/hyperactivity disorder can cause SHB through several mechanisms. Balázs et al. (25) indicated that both disorders, ADHD and SHB, are related to poorer response inhibition and impulsivity that consequently leads to self-mutilation. Moreover, disinhibition could be mediated by coexisting comorbidities, such as affective, anxiety, drug, and alcohol abuse disorders associated with ADHD. Thus, we suggest that disinhibition and impulsivity may lead to performance of NSSI.

### Conclusions and Clinical Implications

1. Self-harm behavior, including NSSI and unintentional self-injury (USI), is a frequent and bothersome symptom in GTS.
2. Current SHB is mainly related to tic severity.
3. Lifetime SHB is mainly associated with OCD and ADHD.
4. The clinical correlates of SHB are likely to be age-dependent. Whereas in children with GTS the main risk factor for SHB is tic severity, in group of adults, anxiety disorder and probably other psychiatric comorbidities attribute to development of SHB.
5. We failed to demonstrate any differences between intentional and accidental SHB, and we assume that tic-related and non–tic-related SHBs seem to be overlapping phenomena, extremely difficult to distinguish.
6. Although per definition SIB must be deliberate, some behaviors in GTS are inadvertent, and that could bias the rate of SIB in the previous studies.

### Limitations

The biggest limitation of our study is the small study sample. The one-time registration study design may have inﬂuenced the prevalence of SHB. Therefore, we cannot determine whether lifetime SHB had repetitive character. There is also a possible referral bias because the patients were evaluated by a movement disorders specialist, and the cases with a more severe psychopathology were referred to psychiatric clinics. Also, recall bias should be taken into consideration. It is also possible that we did not include patients with extreme SHB as those are usually admitted to emergency units or referred to psychiatrists. As mentioned before, we used diﬀerent methods of data collection from children and adults. Most of the clinical information regarding children was provided by their parents, whereas adults reported it themselves. The severity and intentionality of SHB were evaluated only subjectively, as we do not have any objective measurements to differentiate tic-related and non–tic-related SHB. Moreover, some tics could involve also deliberate self-harm as sometimes there may be an urge to harm before the tic. Therefore, future research should also focus on the development of scales or diagnostic criteria that could help to establish the diagnosis of SHB in GTS and divide those patients into subsequent groups.

## Data Availability

The datasets generated for this study are available on request to the corresponding author.

## Ethics Statement

Collecting of clinical data from patients with GTS has been approved by the Ethics Committee of Medical University of Warsaw (KB/2/2007). All subjects gave written informed consent in accordance with the Declaration of Helsinki.

## Author Contributions

PJ conceived and designed the study and acquired data. PJ and NS set up the electronic database. AJ, PJ, and NS analyzed and interpreted the data, performed visualization, and reviewed and edited the manuscript. NS wrote the original draft of the manuscript.

## Conflict of Interest Statement

The authors declare that the research was conducted in the absence of any commercial or financial relationships that could be construed as a potential conflict of interest.
